# The mitochondrial genome of the toothed top shell snail *Monodonta labio* (Gastropoda: Trochidae): the first complete sequence in the subfamily monodontinae

**DOI:** 10.1080/23802359.2019.1711221

**Published:** 2020-01-14

**Authors:** Haiyan Cong, Yixuan Lei, Lingming Kong

**Affiliations:** a Weihai Municipal Hospital, Cheeloo College of Medicine, Shandong University, Shandong, P.R. China;; b Marine College, Shandong University, Shandong, P.R. China

**Keywords:** *Monodonta labio*, mitogenome, phylogeny

## Abstract

The mitochondrial genome (mitogenome) is a powerful tool that is extensively used in genomic and phylogenetic analysis. In this study, the complete mitogenome of the toothed top shell snail (*Monodonta labio*) has been sequenced and annotated for the first time. The complete circular genome is 16,440 bp in length including 13 protein-coding genes, 22 transfer RNA and two ribosomal RNA genes. All of the protein-coding genes use the standard initiation codon ATN and are terminated by the termination codons TAA and TAG. All of the tRNA genes have the typical clover leaf structure, with the exception of the tRNA-Asp, which lacks aTψC arm, and tRNA-Ser(AGN), which lacks a DHU arm. Relatively short intergenic spacers and overlaps are observed in this mitogenome. Our phylogenetic tree shows that *M. labio* is clustered together with other species within Trochidae. The complete mitogenome of *M. labio* provide essential DNA data for evolutionary and phylogenetic analysis of marine gastropods.

Gastropods constitute the most diverse class of the phylum Mollusca, including almost three quarters of the 110,000 or so known species of mollusks. Gastropods have been intensively researched due to their role as intermediate hosts for parasites (Thieltges et al. 2013), and their great economic and ecological value, derived from their widespread use for food and decoration. So far, less than 200 gastropods have had their mitogenomes fully sequenced and published (GenBank: www.ncbi.nlm.nih.gov/genbank), meaning there is still insufficient mitogenomic data to reliably infer the patterns and trends in the genomic evolution of this group. Moreover, because of their high diversity, their systematics are also controversial and under continual revision (Galindo et al. 2016). In this study, to supplement the available genetic data on gastropods, the mitogenome of *Monodonta labio* were sequenced. *Monodonta labio* is conspicuous components of intertidal rocky shores, with a wide Indo-Pacific distribution (Huang 1994). The obvious distinguishing features of this snail are with a tooth in its shell opening.

We here first report the first mitogenome of *M. labio* collected from the coastal rock of Jing Shui bay, Weihai, Shandong Province, P.R. China (E122°7′17.04″, N37°32′58.56″). The specimen and its DNA were stored at the Laboratory of Molecular Biology, Marine College, Shandong University (Weihai) (KC-dancl-002). The complete mitogenome of the specimen was determined using Sanger sequencing. The mitogenome has been deposited in the NCBI GenBank under the accession numbers, MK240320. The mitogenome of *M. labio* is 16,440 bp in length. The heavy strand codes 16 genes, whereas the others are coded by the light strand. This mitogenome contains three gene overlaps and 32 intergenic spacers (IGSs). The first overlap, 5 bp in length, is located between tRNA-Lys and tRNA-Ala. The second (7 bp) is between tRNA-Leu and 16S rRNA, and the third (1 bp) between tRNA-Trp and tRNA-Gln. The IGSs range in length from 1 to 418 bp, with a total length of 1039 bp. The overall A + T content of *M. labio*is 59.2%. The AT skew (0.064) is slightly positive, which means that there is more A than T, whereas the GC skew (−0.314) is negative, indicating a higher content of C than G.

Twenty-nine species with complete 13 PCGs (excluding termination codons) were used in phylogenetic analyses, *Rapana venosa* used as outgroup. Each PCG was aligned individually with codon-based multiple alignments using MUSCLE v3.8.31 (Edgar 2004). Alignments of individual genes were then concatenated as a combined matrix. Maximum-likelihood (ML) tree was inferred using IQ-tree (Nguyen et al. 2015) using the models detected with ModelFinder (Kalyaanamoorthy et al. 2017), and node confidence was assessed with 1000 ultrafast bootstrap replicates. In the ML tree of concatenated nucleotide sequences from 13 PCGs, *M. labio* is clustered together with other species within Trochidae (Figure 1). Our topology of the phylogenetic tree is consistent with the results of previous molecular studies (Uribe et al. 2016, 2017).

**Figure 1.**
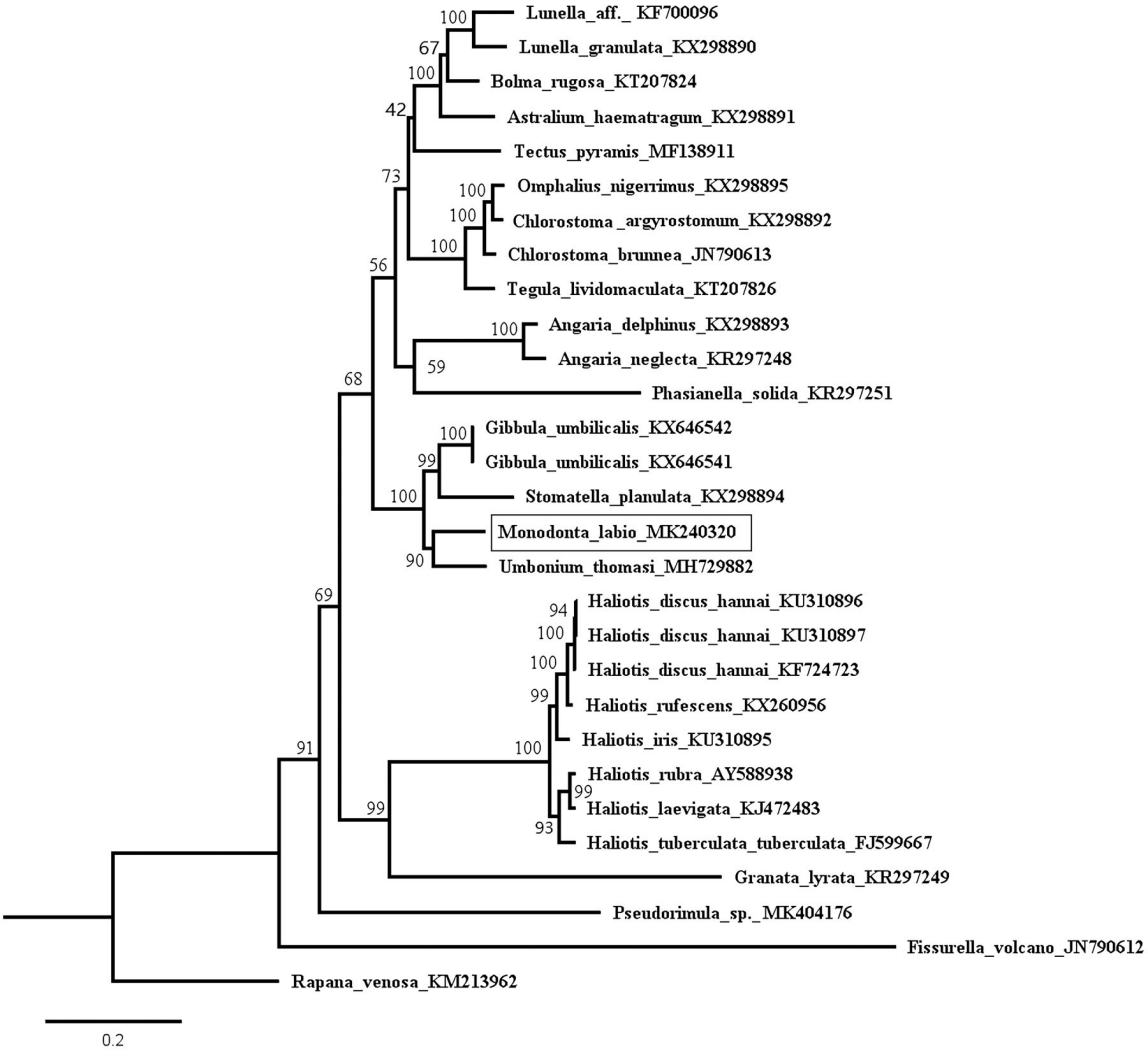
Maximum-likelihood tree of Vetigastropoda based on the combined dataset of first and second codon positions of 13 PCGs. Numbers above branches indicate maximum-likelihood bootstrap support values. The best model for this combined dataset was GTR + F+I + G4.

## References

[CIT0001] Edgar RC. 2004. MUSCLE: multiple sequence alignment with high accuracy and high throughput. Nucleic Acids Res. 32(5):1792–1797. 15034147 10.1093/nar/gkh340 PMC390337

[CIT0002] Galindo LA, Puillandre N, Utge J, Lozouet P, Bouchet P. 2016. The phylogeny and systematics of the Nassariidae revisited (Gastropoda, Buccinoidea). Mol Phylogenet Evol. 99:337–353. 27012605 10.1016/j.ympev.2016.03.019

[CIT0003] Huang Z. 1994. Marine species and their distributions in China seas. Beijing: China Ocean Press.

[CIT0004] Kalyaanamoorthy S, Minh BQ, Wong TKF, Haeseler A, von Jermiin LS. 2017. ModelFinder: fast model selection for accurate phylogenetic estimates. Nat Methods. 14(6):587–589. 28481363 10.1038/nmeth.4285 PMC5453245

[CIT0005] Nguyen L-T, Schmidt HA, von Haeseler A, Minh BQ. 2015. IQ-TREE: a fast and effective stochastic algorithm for estimating maximum-likelihood phylogenies. Mol Biol Evol. 32(1):268–274. 25371430 10.1093/molbev/msu300 PMC4271533

[CIT0006] Thieltges D. W, Marcogliese D. J, Blanar C. A, Poulin R. 2013. Trematode prevalence-occupancy relationships on regional and continental spatial scales in marine gastropod hosts. Mar Ecol Prog Ser. 490:147–154.

[CIT0007] Uribe J. E, Kano Y, Templado J, Zardoya R. 2016. Mitogenomics of Vetigastropoda: insights into the evolution of pallial symmetry. Zool Scr. 45(2):145–159.

[CIT0008] Uribe J. E, Williams S. T, Templado J, Abalde S, Zardoya R. 2017. Denser mitogenomic sampling improves resolution of the phylogeny of the superfamily Trochoidea (Gastropoda: Vetigastropoda). J Mollus Stud. 83(1):111–118.

